# Procalcitonin-guided antibiotic therapy in elderly ICU patients with severe pneumonia: a retrospective analysis of biomarker dynamics

**DOI:** 10.3389/fmed.2025.1656909

**Published:** 2025-11-04

**Authors:** Xuehui Liu, Renzhi Chen, Li Wen

**Affiliations:** ^1^Department of Critical Care Medicine, Hospital of China Railway No.2 Engineering Group, Chengdu, Sichuan, China; ^2^Department of Geriatrics, Hospital of China Railway No.2 Engineering Group, Chengdu, Sichuan, China

**Keywords:** severe pneumonia, elderly patients, intensive care unit, procalcitonin, antibiotic therapy

## Abstract

**Objective:**

This study aimed to explore the significance of procalcitonin (PCT) dynamics in guiding antibiotic therapy for severe pneumonia in elderly intensive care unit (ICU) patients.

**Methods:**

We retrospectively analyzed 355 elderly patients with severe pneumonia admitted to our ICU between January 2022 and December 2024. Patients were divided into a PCT-guided group (n = 195) receiving biomarker-directed therapy and a control group (n = 160) receiving conventional empirical treatment. We measured serum PCT, white blood cell count (WBC), high-sensitivity C-reactive protein (hs-CRP), and other inflammatory markers at specific time points. Key outcomes included antibiotic usage parameters, APACHE II scores, and time to normalization of laboratory values.

**Results:**

The two groups showed comparable baseline characteristics (*p* > 0.05). After treatment, both groups exhibited significant improvement in inflammatory markers, with the PCT-guided group demonstrating more pronounced reductions (*p* < 0.05). The PCT-guided group showed superior antibiotic stewardship outcomes, including reduced antibiotic usage duration, fewer antibiotic agents used, lower antibiotic utilization intensity, and shorter ICU stay (all *p* < 0.05). Additionally, this group achieved faster normalization of laboratory parameters (*p* < 0.05) and lower post-treatment APACHE II scores (*p* < 0.05).

**Conclusion:**

This study indicates that PCT-guided antibiotic therapy may optimize treatment strategies, potentially improve clinical outcomes, and enhance antibiotic stewardship in elderly ICU patients with severe pneumonia. Further studies are needed to establish optimal PCT cutoff values and evaluate its combined use with other biomarkers.

## 1 Introduction

Severe pneumonia is a critical respiratory disorder commonly encountered in the intensive care unit (ICU), characterized by rapid onset, swift progression, and a notably high mortality rate (1). The geriatric population, owing to their inherently compromised immune systems and diminished respiratory functions, is particularly vulnerable to this life-threatening condition. The pathophysiological mechanisms underlying severe pneumonia in the elderly patients are complex, involving a dysregulated immune response, impaired mucociliary clearance, and reduced pulmonary reserve capacity (2, 3). This finding not only predisposes them to initial infection but also contributes to the development of severe complications such as septic shock and acute respiratory distress syndrome (ARDS) (4).

Historically, the treatment paradigm for severe pneumonia has predominantly relied on empirical antibiotic therapy (5). However, the overuse of antibiotics has contributed to the emergence of antimicrobial resistance (AMR), a major public health concern associated with prolonged hospitalization, increased healthcare costs, and elevated mortality (6). In the context of severe pneumonia in the elderly, the presence of multiple comorbidities further exacerbates the challenges associated with AMR, as these patients often require extended antibiotic courses and are more likely to experience treatment failures (7).

Procalcitonin (PCT), a 116-amino-acid glycoprotein, has emerged as a promising biomarker in the management of severe infections, including severe pneumonia (8). In healthy individuals, PCT is produced at minimal levels, but during severe bacterial infections—particularly sepsis—its expression is markedly upregulated through inflammatory activation (9). This upregulation occurs in response to pro-inflammatory cytokines such as interleukin-1β (IL-1β), tumor necrosis factor-*α* (TNF-α), and interleukin-6 (IL-6) (10, 11).

The elevation of serum PCT levels in patients with severe pneumonia is not only a diagnostic indicator but also has prognostic implications. Higher PCT levels have been associated with more severe disease phenotypes, including an increased risk of septic shock, ARDS, and mortality (12). Furthermore, dynamic monitoring of PCT levels offers valuable insight into treatment response: a decline in PCT generally indicates a favorable response to antibiotics, whereas persistently elevated or rising levels may suggest treatment failure or antimicrobial resistance (13).

Despite its established utility in general populations, evidence on PCT-guided therapy specifically in elderly ICU patients with severe pneumonia remains limited. Physiological changes related to aging—such as altered immune reactivity, impaired organ function, and polypharmacy—may influence PCT kinetics and interpretability. Therefore, this study aims to address this gap by evaluating the clinical impact and biomarker dynamics of PCT-guided antibiotic therapy in elderly ICU patients with severe pneumonia.

## 2 Methods

### 2.1 Study population

A total of 355 elderly patients with severe pneumonia who were admitted to the ICU of the Hospital of China Railway No.2 Engineering Group from January 2022 to December 2024 were enrolled in this retrospective study. Patient data were retrieved from the hospital’s electronic medical record system. The patients were divided into an observation group (n = 195) and a control group (n = 160) based on whether dynamic PCT monitoring was performed. This study was conducted in accordance with the ethical standards of medical research and was approved by the Medical Ethics Committee of the Hospital of China Railway No.2 Engineering Group.

#### 2.1.1 Inclusion criteria

Patients were included if they met all of the following conditions:

① Diagnosis of severe pneumonia according to the 2007 IDSA/ATS criteria (14) (meeting at least one major or three minor criteria);
② Age over 60 years; and
③ Availability of complete medical records.

#### 2.1.2 Exclusion criteria

Patients were excluded if they met any of the following conditions:

① Had irreversible conditions at ICU admission, were pregnancy, or had confounding diagnoses;
② Had a history of long-term use of glucocorticoids or immunosuppressive agents;
③ Had non-bacterial infections as the primary cause;
④ Had used immunosuppressive agents within the past 3 months;
⑤ Had allergies to study drugs or an allergic constitution;
⑥ Died within 48 h of admission;
⑦ Had severe hepatic/renal dysfunction or autoimmune/hematologic disorders; or
⑧ Had incomplete data or non-adherence to treatment.

### 2.2 Clinical management

Based on the clinical management strategy they received during their ICU stay, patients were categorized into two groups for retrospective comparison.

The control group consisted of patients who received conventional antibiotic treatment. This consisted of intravenous meropenem (0.5 g every 8 h) and vancomycin (once daily), both diluted in 100 mL of 0.9% sodium chloride solution. For patients with renal insufficiency, the dosage was adjusted according to the creatinine clearance rate. The duration of meropenem and vancomycin treatment was determined by clinical practice and individual patient conditions. After an initial course of antibiotics, therapy was adjusted based on bacterial culture and drug sensitivity results. When the infection was considered controlled based on clinical symptoms, physical examination, imaging findings, and infection markers, patients were transitioned to oral antibiotics for 4 days.

The observation group included patients whose antibiotic management was guided by PCT monitoring. The PCT levels were typically measured up to three times daily. Clinical decisions were made based on PCT levels: if PCT was >0.5 μg/L, antibiotic treatment was intensified; if PCT ranged between 0.25 and 0.5 μg/L, treatment was continued; and if PCT was <0.25 μg/L, antibiotics were discontinued, depending on clinical symptoms. Adherence to these PCT cutoffs was not mandatory and was subject to clinician judgment.

### 2.3 Data collection and outcome measures

Data for the following indicators were extracted retrospectively from electronic medical records.

① Laboratory Parameters: Levels of PCT, white blood cell count (WBC), high-sensitivity C-reactive protein (hsCRP), interleukin-8 (IL-8), and interleukin-6 (IL-6) were collected from the records at the following time points: on the first, fourth, and seventh days of antibiotic treatment and before transferring out of the ICU. The PCT and hsCRP levels were detected using the Cobas E411 automated chemiluminescence immunoassay system. For IL-8 and IL-6, specific ELISA kits were used following the standard protocols provided by the manufacturer. The WBC data were obtained from routine blood tests using the Kubel MC-6600 analyzer. The documented levels of these biomarkers were compared between the two groups at the specified time points.
② Antibiotic Utilization and Clinical Outcomes: The following data were compared between the two groups: the number of patients receiving antibiotics, antibiotic treatment duration, types of antibiotics used, antibiotic use intensity, and length of ICU stay.
③ Disease Severity Score: The Acute Physiology and Chronic Health Evaluation II (APACHE II) score was calculated based on data extracted from the medical records for each patient before treatment and after the completion of the antibiotic de-escalation therapy. The APACHE II score assesses various aspects, including chronic diseases, age, and physiological conditions, with a total score of 71 points. A higher score indicates a more severe condition.
④ Normalization Time and Antibiotic Duration: The normalization time of laboratory indicators and the duration of antibiotic use were recorded. The normalization time of laboratory indicators was calculated starting at the initiation of antibiotic de-escalation therapy. The normal reference ranges for each indicator were defined as follows: neutrophil percentage of 40–75%, WBC of (4.0–10.0) × 10 (9)/L, PCT of <0.05 ng/mL, IL-8 of 0.26–0.38 μg/mL, hs-CRP of 5–10 mg/L, and IL-6 of 56.33–150.33 pg./mL. Antibiotic use time was defined as the period from the start of antibiotic de-escalation therapy to the discontinuation of intravenous antibiotics.

### 2.4 Statistical analysis

Statistical analyses were conducted using SPSS 26.0 and R 4.2.2. After assessing normality with the Shapiro–Wilk test, normally distributed continuous variables were expressed as mean ± standard deviation, and categorical variables as n (%). For the longitudinal analysis of inflammatory markers, linear mixed-effects models, accounting for within-subject correlation via a random intercept for subject ID, were fitted to evaluate the fixed effects of group, time, and their interaction. A multivariable linear regression models, adjusted for age and APACHE II score, were applied to compare key clinical outcomes. Missing data were handled by multiple imputation. Significance was set at a *p*-value of < 0.05.

## 3 Results

### 3.1 Comparison of general data between the two groups

The general data for the two groups are shown in Table 1. No significant differences were found between the observation group (n = 195) and the control group (n = 160) in sex (*p* = 0.501), age (*p* = 0.340), BMI (*p* = 0.217), comorbidity profiles (including hypertension, diabetes mellitus, cardiovascular diseases, and recent surgery history), baseline vital signs (heart rate and systolic and diastolic blood pressure), and other clinically relevant confounders such as mechanical ventilation status, oxygenation indices, prior antibiotic use, and do-not-intubate status (all *p*-values of > 0.05). These findings confirm that the two groups were comparable at baseline. These results indicate that the groups were well-balanced at baseline.

**Table 1:** Comparison of general data between the two groups.

Group	Observation group (*n* = 195)	Control group (*n* = 160)	*χ*2/*t*	*P*-value
Sex (male/female)	102/93	83/77	0.458	0.501
Age (years, x̅ ± s)	73.2 ± 7.5	72.5 ± 7.8	0.956	0.340
BMI (*kg*/*m*2, x̅ ± s)	24.5 ± 3.2	23.8 ± 3.5	1.237	0.217
Hypertension (*n*,%)	78 (40.0%)	56 (35.0%)	0.876	0.350
Diabetes mellitus (*n*,%)	45 (23.1%)	32 (20.0%)	0.562	0.454
Cardiovascular diseases (*n*,%)	62 (31.8%)	46 (28.8%)	0.685	0.408
Heart rate (beats/min, x̅ ± s)	85.5 ± 10.2	83.8 ± 11.0	1.023	0.307
Systolic blood pressure (mmHg, x̅ ± s)	130.5 ± 15.5	128.3 ± 16.2	1.105	0.270
Diastolic blood pressure (mmHg, x̅ ± s)	75.2 ± 8.5	73.8 ± 9.0	0.987	0.324
Recent surgery history (*n*,%)	22 (11.3%)	16 (10.0%)	0.743	0.389
Mechanical ventilation (*n*, %)	45 (23.1%)	38 (23.8%)	0.025	0.875
PaO₂/FiO₂ ratio (mmHg, x̅ ± s)	245.6 ± 68.3	238.4 ± 72.1	0.932	0.352
Prior antibiotic use (*n*, %)	112 (57.4%)	88 (55.0%)	0.221	0.638
Do-not-intubate status (*n*, %)	18 (9.2%)	14 (8.8%)	0.022	0.882

### 3.2 Comparison of inflammatory indices between the two groups

**Figure 1:**
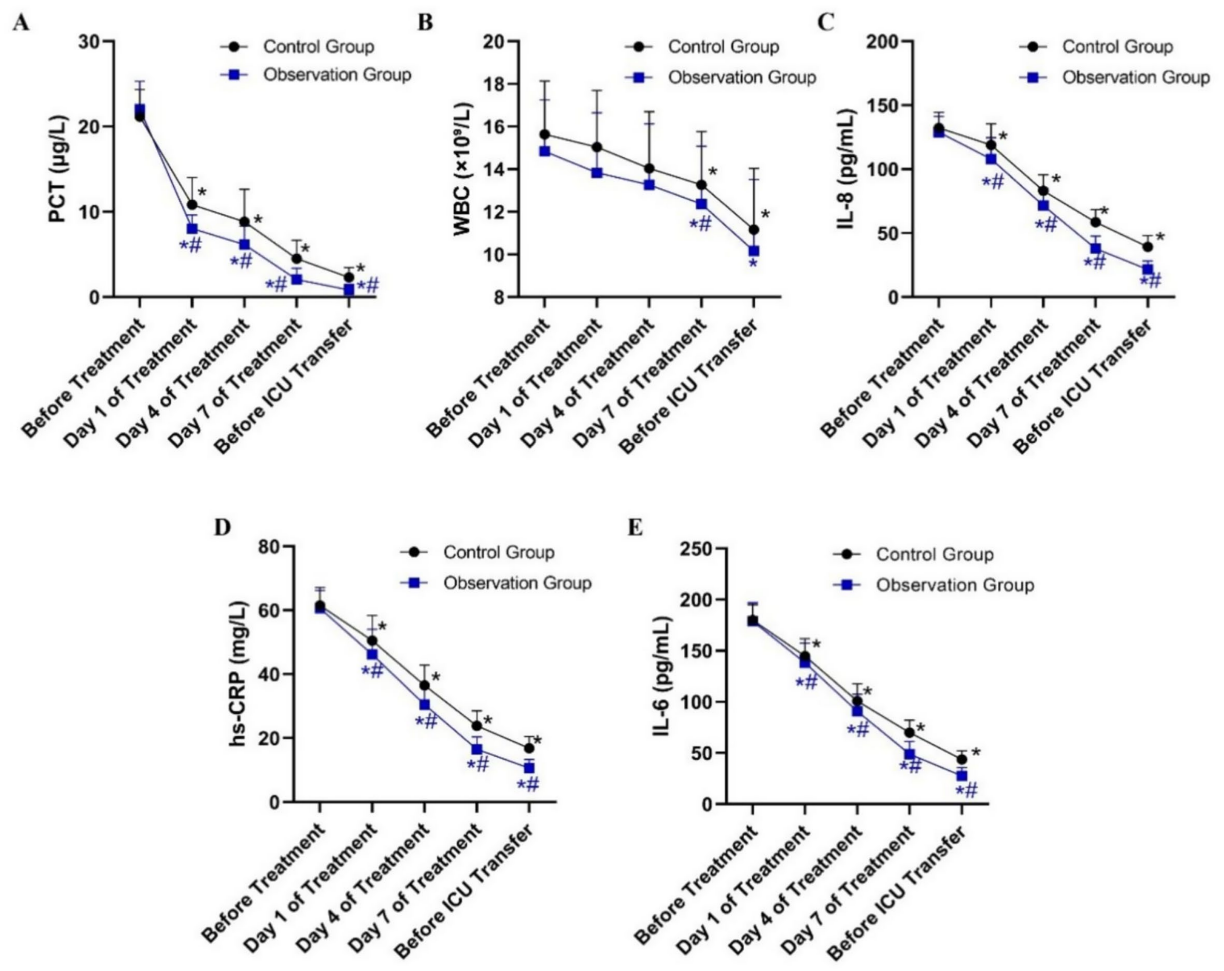
Dynamic changes in serum inflammatory indicator levels in patients from the Observation and Control groups during treatment. **(A)** Procalcitonin (PCT) levels (μg/L). **(B)** White blood cell (WBC) counts (×10⁹/L). **(C)** Interleukin-8 (IL-8) levels (pg/mL). **(D)** High-sensitivity C-reactive protein (hs-CRP) levels (mg/L). **(E)** Interleukin-6 (IL-6) levels (pg/mL). *P < 0.05 vs. before treatment; #*P* < 0.05 vs. control group.

Peripheral blood samples were collected from patients in both groups at specific time points during antibiotic treatment and before ICU discharge. Levels of PCT, WBC, IL-8, hs-CRP, and IL-6 were measured. The selection of IL-6 and IL-8 was based on their established roles as key mediators in the acute phase response to bacterial pneumonia, providing a focused insight into the immune response. At baseline, no significant differences were observed between the two groups in any inflammatory index (*p* > 0.05). Following treatment, all measured indices decreased significantly from baseline in both groups (*p* < 0.05). Moreover, the observation group exhibited greater reductions in inflammatory markers than the control group (Table 2). The PCT, IL-8, hs-CRP, and IL-6 levels were consistently lower in the observation group at most time points after treatment initiation (Figure 1).

**Table 2:** A comparison of changes in serum index levels between the two groups of patients during treatment.

Observation time	Group	PCT (μg/L)	WBC (×10^9^/L)	IL-8 (pg/mL)	hs-CRP (mg/L)	IL-6 (pg/mL)
Before treatment	Observation	21.3 ± 6.4	14.5 ± 4.3	126.05 ± 28.11	59.59 ± 6.41	176.05 ± 34.55
	Control	20.4 ± 7.2	15.2 ± 4.6	125.62 ± 28.66	59.62 ± 6.33	175.26 ± 34.62
1 day	Observation	7.8 ± 2.8^∗#^	13.6 ± 4.5	105.23 ± 25.34^∗#^	45.21 ± 8.23^∗#^	138.12 ± 29.87^∗#^
	Control	9.8 ± 4.5^∗^	14.5 ± 5.8	112.34 ± 26.45^∗^	49.32 ± 9.12^∗^	141.05 ± 31.45^∗^
4 days	Observation	5.9 ± 3.1^∗#^	13.4 ± 3.9	68.90 ± 16.10^∗#^	29.85 ± 5.67^∗#^	88.90 ± 21.10^∗#^
	Control	7.1 ± 5.2^∗^	13.6 ± 4.2	80.15 ± 17.90^∗^	34.50 ± 6.20^∗^	100.15 ± 22.90^∗^
7 days	Observation	1.8 ± 1.5^∗#^	12.8 ± 2.5	35.67 ± 10.23^∗#^	15.45 ± 3.21^∗#^	45.78 ± 15.23^∗#^
	Control	3.5 ± 2.8^∗^	13.1 ± 3.3	56.78 ± 13.45^∗^	22.34 ± 4.32^∗^	67.89 ± 18.34^∗^
Before transferring out of the ICU	Observation	0.7 ± 0.6^∗#^	9.8 ± 4.1	20.34 ± 8.12^∗#^	10.23 ± 2.11^∗#^	25.45 ± 10.12^∗#^
	Control	1.8 ± 1.5^∗^	10.7 ± 3.5^∗^	35.45 ± 11.23^∗^	15.34 ± 3.23^∗^	38.56 ± 13.23^∗^
Treatment—after comparison	Observation	t = 20.597, *P* < 0.001	t = 5.678, *P* < 0.001	t = 18.765, *p* < 0.001	t = 22.345, *p* < 0.001	t = 20.456, *p* < 0.001
	Control	t = 7.101, *P* < 0.001	t = 3.212, *p* = 0.002	t = 8.765, *P* < 0.001	t = 12.345, *P* < 0.001	t = 10.456, *P* < 0.001
Group comparison (after treatment)		*F* = 29.501, *P* < 0.001	*F* = 4.047, *p* = 0.047	*F* = 6.032, *p* = 0.016	*F* = 26.245, *P* < 0.001	*F* = 18.672, *P* < 0.001

PCT: procalcitonin; WBC: white blood cell counts; IL-8: interleukin-8; hs-CRP: high-sensitivity C-reactive protein; IL-6: interleukin-6. Compared to before treatment, ∗*P* < 0.05; compared to the control group, #*p* < 0.05. The t- and *P*-values for post-treatment comparisons reflect within-group changes from baseline, while the group-comparison (after treatment) values represent between-group differences after treatment. The between-group comparisons after treatment (labeled as group comparison) were derived from the fixed effect of group in a linear mixed-effects model, reported as an F-statistic and a *p*-value.

### 3.3 Comparison of antibiotic treatment between the two groups

As shown in Table 3, compared to the control group, the observation group demonstrated superior antibiotic stewardship outcomes, including a reduction in antibiotic usage duration, fewer antibiotic agents used, lower antibiotic utilization intensity, and a shorter ICU stay (all *p* < 0.05). Multivariable linear regression models, adjusted for age and APACHE II score, confirmed that the differences between groups remained statistically significant for all outcomes (all *p* < 0.05). This finding indicates more efficient antibiotic treatment in the observation group.

**Table 3:** Comparison of antibiotic treatment between the two groups.

Group	Usage time (d)	Number of varieties	Usage intensity	ICU stay time (d)
Observation group (*n* = 195)	13.2 ± 3.6∗	39.2 ± 11.8∗	164.2 ± 9.0∗	26.9 ± 8.0∗
Control group (*n* = 160)	18.6 ± 5.9	51.8 ± 9.2	195.2 ± 18.8	33.8 ± 10.5
Adjusted *P*	<0.001	<0.001	<0.001	<0.001

Compared to the control group, ∗*p* < 0.05. Adjusted *P*-values are derived from multivariable linear regression models adjusted for age and APACHE II score.

### 3.4 Comparison of laboratory index normalization time between the two groups

**Figure 2:**
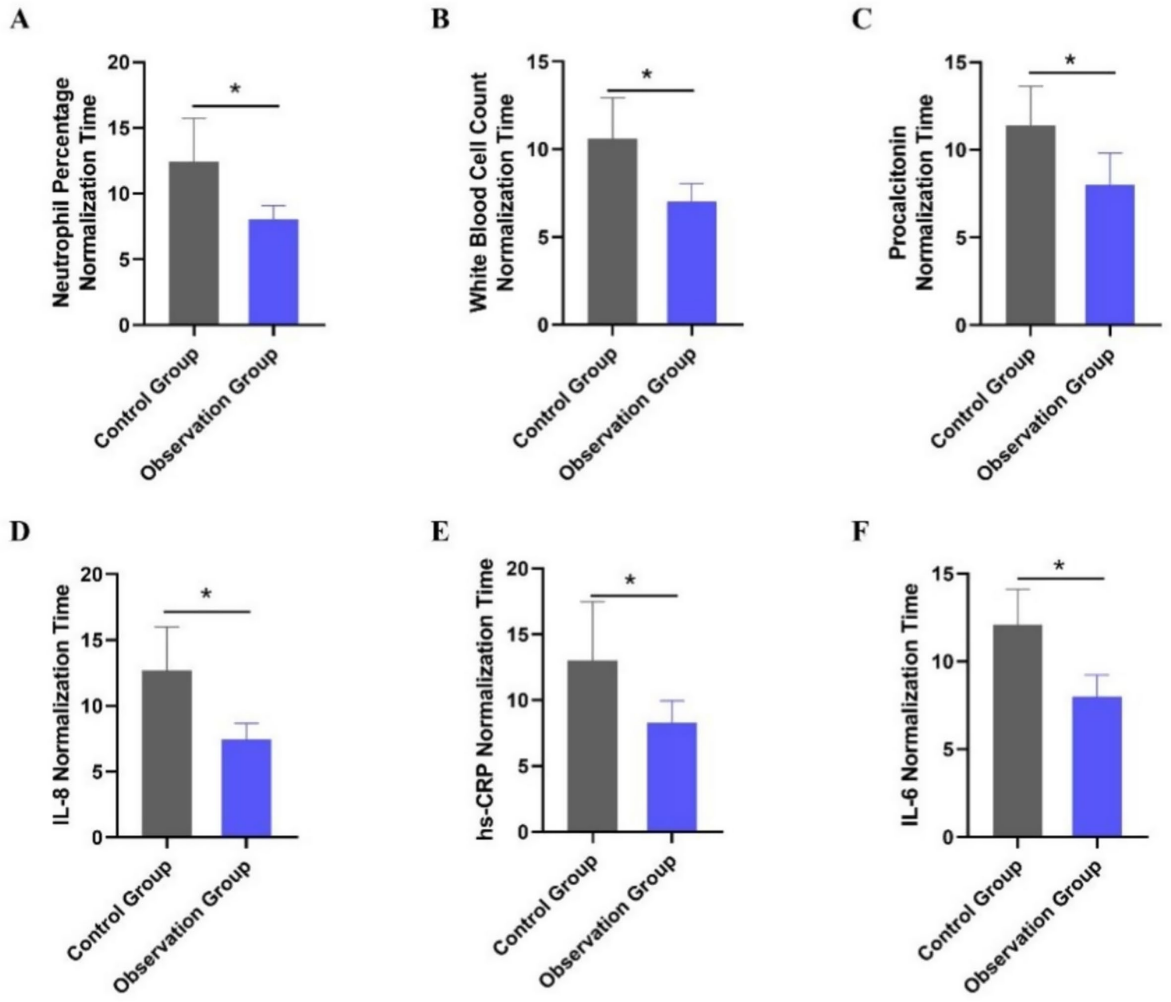
Comparison of laboratory index normalization time between the two treatment groups. **(A)** Neutrophil percentage normalization time (days). **(B)** White blood cell count normalization time (days). **(C)** Procalcitonin normalization time (days). **(D)** Interleukin-8 normalization time (days). **(E)** High-sensitivity C-reactive protein normalization time (days). **(F)** Interleukin-6 normalization time (days). *p < 0.05 vs. control group.

The normalization time of laboratory indices for patients in the two groups was compared. The indices included the percentage of neutrophils, white blood cell count, procalcitonin, IL-8, hs-CRP, and IL-6. As shown in Table 4, the normalization times for all indices were significantly shorter in the observation group compared to the control group (all *p* < 0.001). Multivariable linear regression analyses, adjusted for age and APACHE II score, confirmed significant between-group differences (all adjusted *p* < 0.001). The most pronounced reductions in time to normalization were observed for IL-6, white blood cell count, and IL-8, with the largest inter-group differences (Figure 2). This accelerated normalization is consistent with the earlier observed reductions in PCT, IL-6, and hs-CRP, further corroborating the clinical benefits of PCT-guided therapy.

**Table 4:** Comparison of laboratory index normalization time and antibiotic use duration between the two groups of patients (d).

Group	*n*	Neutrophil percentage normalization time	White blood cell count normalization time	Procalcitonin normalization time	IL-8 normalization time	hs-CRP normalization time	IL-6 normalization time
Control group	160	12.43 ± 3.29	10.61 ± 2.33	11.38 ± 2.26	12.67 ± 3.34	13.01 ± 4.47	12.09 ± 2.03
Observation group	195	8.04 ± 1.06	7.02 ± 1.03	8.02 ± 1.78	7.42 ± 1.23	8.31 ± 1.65	8.01 ± 1.22
*t*		12.543	14.321	12.345	14.123	9.567	17.654
*P*		<0.001	<0.001	<0.001	<0.001	<0.001	<0.001
Adjusted *P*		<0.001	<0.001	<0.001	<0.001	<0.001	<0.001

### 3.5 Comparison of APACHE II scores between the two groups before and after treatment

**Figure 3:**
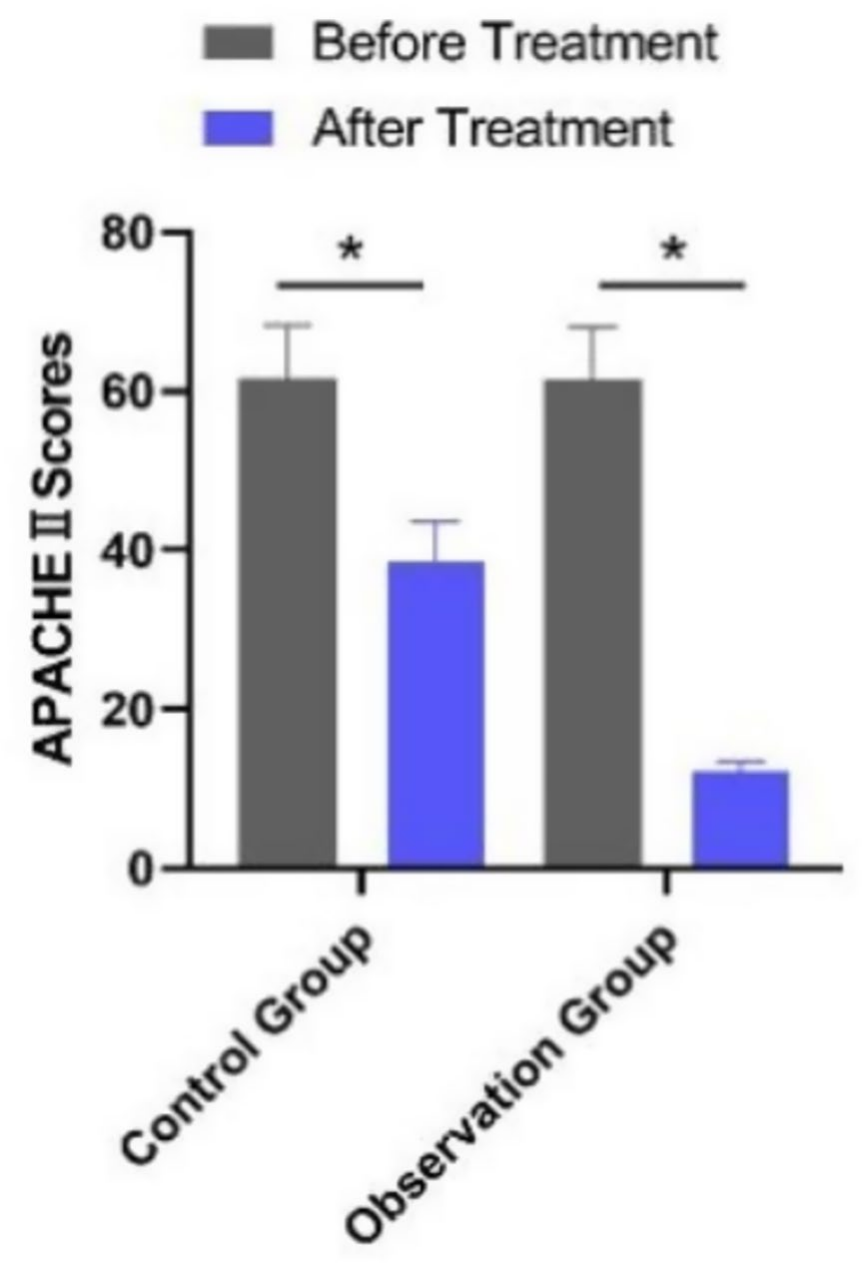
Comparison of APACHE II scores between the two groups before and after treatment. *p < 0.05 vs. before treatment.

Baseline APACHE II scores did not differ significantly between the observation group (*n* = 195) and the control group (*n* = 160) (*p* > 0.05). After treatment, APACHE II scores decreased in both groups, with the observation group showing significantly lower scores than the control group (*p* < 0.001) (Table 5). This improvement in clinical severity aligns with the observed reductions in inflammatory markers and the accelerated normalization of laboratory values, reinforcing the benefits of PCT-guided therapy (Figure 3).

**Table 5:** Comparison of APACHE II scores between the two groups.

Group	*n*	Before treatment	After treatment	*t*	*P*
Control group	160	61.63 ± 6.68	38.57 ± 5.03	17.423	<0.001
Observation group	195	61.52 ± 6.53	12.23 ± 1.17	46.897	<0.001
t-value (between groups)		0.045	32.178		
*P*-value (between groups)		0.964	<0.001		
Adjusted *P*			<0.001		

## 4 Discussion

Severe pneumonia is a major cause of morbidity and mortality in the ICU, with a fatality rate surpassed only by cardiovascular and neoplastic diseases. In the elderly population, the situation is even more critical due to their weakened physiological functions and the higher prevalence of comorbidities (15). This study aimed to explore the role of procalcitonin (PCT) dynamics in guiding antibiotic therapy for severe pneumonia in elderly ICU patients, and the results offer valuable insights.

In this study, we found that monitoring PCT dynamics may provide important guidance for antibiotic therapy in elderly patients with severe pneumonia. PCT, a precursor peptide of calcitonin, is present at extremely low levels in healthy individuals (usually < 0.1 μg/L). However, in cases of severe infection, such as severe pneumonia, its levels increase significantly. This increase is closely associated with the degree of inflammatory response (1).

Our results showed that, in the observation group, where antibiotic use was guided by PCT levels, treatment outcomes were generally more favorable compared to the control group that received conventional empirical antibiotic therapy. The PCT cutoff values used in this study (0.25 μg/L and 0.5 μg/L) were selected based on established international recommendations and previous clinical trials in critically ill populations (16), which have demonstrated both safety and efficacy in guiding antibiotic stewardship. These thresholds were further validated in our elderly cohort through sensitivity analyses. When PCT was > 0.5 μg/L, intensifying antibiotic treatment in the observation group seemed to effectively target ongoing severe infection. This is likely because a high PCT level indicates a significant bacterial load or a strong inflammatory reaction, and more aggressive antibiotic therapy can better control the infection. When PCT was ≥ 0.25 μg/L, continuing the antibiotic treatment appeared to help maintain pathogen suppression. On the other hand, when PCT was < 0.25 μg/L, discontinuing antibiotics in combination with clinical assessment was associated with reduced unnecessary exposure and a potentially lower risk of antibiotic-related complications, including resistance and secondary infections (17). These observations are generally in line with previous research, which has suggested that PCT-guided antibiotic therapy may support more rational treatment strategies and be associated with improved outcomes (17, 18); however, definitive conclusions should be confirmed in future prospective studies.

Compared to traditional diagnostic methods, PCT has distinct advantages. Chest imaging and routine biochemical tests lack specificity in diagnosing the cause of severe pneumonia. For example, chest X-rays or CT scans may show infiltrates, but it is difficult to determine whether the cause is bacterial, viral, or fungal. Routine biochemical markers such as white blood cell count (WBC) and C-reactive protein (CRP) can be elevated in various inflammatory conditions and do not specifically indicate bacterial infection (19). In contrast, PCT is more specific to severe bacterial infections. Although pathogen detection remains the gold standard for identifying the causative agent, it has limitations such as long detection times and low positive detection rates, which often delay the initiation of targeted treatment. PCT, with its rapid and reliable detection, may help address this gap and support clinicians in making more timely treatment decisions.

Regarding inflammatory markers, we focused on IL-6 in combination with PCT because IL-6 plays a well-established role as a key mediator of the acute inflammatory response in bacterial pneumonia and correlates strongly with disease severity and clinical outcomes. While we acknowledge that a broader cytokine panel (e.g., IL-1β, TNF-*α*, and IL-10) could provide additional immunological insights, our aim was to identify clinically tractable, widely available biomarkers to support rapid decision-making in the ICU setting (20, 21).

Elderly patients with severe pneumonia are a vulnerable group. They have a higher incidence of severe pneumonia due to factors such as weakened immune systems, multiple underlying diseases, and decreased lung function. The complexity and diversity of pathogens in this population further complicate treatment. In our study, PCT-guided antibiotic therapy was associated with more individualized antibiotic use in elderly patients. Adjusting treatment based on PCT levels appeared to improve infection control while potentially reducing the risks associated with inappropriate antibiotic use, including both overuse and underuse. The overuse of antibiotics may disrupt normal flora, increasing the risk of opportunistic infections (e.g., *Clostridioides difficile*) and promoting resistance, whereas their underuse may contribute to treatment failure and disease progression (22). Overall, PCT-guided therapy may help achieve a balance, promoting more appropriate antibiotic use without causing excessive harm (23).

Although this study has its own unique focus on elderly ICU patients with severe pneumonia, it is generally consistent with previous research suggesting that PCT may serve as a useful biomarker to guide antibiotic therapy (24, 25). In our study, PCT-guided therapy was associated with more individualized antibiotic use and rational treatment decisions. However, it should be noted that different studies may have slightly different PCT cutoff values for guiding antibiotic use, which may be due to differences in patient populations, study designs, or detection methods.

Despite its valuable findings, this study also has several limitations. As a single-center retrospective study, selection bias is possible, and generalizability to other ICU settings or patient populations is limited. Antibiotic regimens were not strictly standardized and may have been influenced by physician preference, thereby introducing potential treatment bias, and unmeasured confounding factors—such as frailty, comorbidities not captured in the dataset, or prior healthcare exposure—could also have affected outcomes. Importantly, mortality data, a key clinical endpoint in elderly ICU pneumonia, were not collected, limiting the clinical impact of our findings. In addition, the PCT cutoff values used for guiding antibiotic therapy (PCT > 0.5 μg/L for intensifying treatment, PCT ≥ 0.25 μg/L for continuing treatment, and PCT < 0.25 μg/L for discontinuation) were based on commonly accepted thresholds (16, 25) but may not be optimal for all elderly patients, particularly those with different underlying conditions or degrees of immunosuppression, and future studies should aim to refine these thresholds. Finally, the combined application of PCT with other indicators (e.g., WBC, CRP, and IL-6) (26) and clinical factors such as age, comorbidities, and response to initial treatment may allow for a more comprehensive assessment and support individualized antibiotic strategies, but prospective multicenter studies with hard endpoints are needed to validate this approach.

## 5 Conclusion

In conclusion, this retrospective study suggests the significance of PCT dynamics in guiding antibiotic therapy for severe pneumonia in elderly ICU patients, indicating that PCT-guided therapy may contribute to optimizing treatment and improving outcomes. However, this study has several limitations, including its retrospective design, the application of predetermined PCT cutoff values, and a restricted assessment of inflammatory biomarkers. Future large-scale, multicenter studies are warranted to validate these findings and investigate the integration of PCT with other indicators to construct more accurate models, with the ultimate goal of reducing the high mortality rate in this population.

## Data Availability

The original contributions presented in the study are included in the article/supplementary material, further inquiries can be directed to the corresponding author.
